# Long non-coding RNA HOTAIRincreased mechanical stimulation-induced apoptosis by regulating microRNA-221/BBC3 axis in C28/I2 cells

**DOI:** 10.1080/21655979.2021.2003129

**Published:** 2021-12-07

**Authors:** Tiansheng Zheng, Jishang Huang, Jinliang Lai, Qingluo Zhou, Tong Liu, Qiang Xu, Guanglin Ji, Yongjun Ye

**Affiliations:** a Department of Orthopedics, The First Affiliated Hospital of Gannan Medical University, Ganzhou, China; b Department of Emergency, The First Affiliated Hospital of Gannan Medical University, Ganzhou, China

**Keywords:** Osteoarthritis, mechanical stimulation, apoptosis, Hotair, miR-221, BBC3

## Abstract

Abnormal mechanical stimulation contributes to articular cartilage degeneration and osteoarthritis (OA) development. Many long noncoding RNAs (lncRNAs) are involved in mechanical force-induced cartilage degeneration. LncRNA HOTAIR (HOTAIR) has been demonstrated to increase osteoarthritis progression. However, the roles of HOTAIR in mechanical stimulation-treated chondrocytes are still unclear. In this study, we found that mechanical stimulation significantly induced apoptosis in C28/I2 cells. In addition, the expression of HOTAIR was up regulated and the expression of miR-221 was down regulated. Knockdown of HOTAIR effectively ameliorated cell apoptosis induced by mechanical stimulation. HOTAIR could interact with miR-221, which targeted to degrade BBC3. Overexpression of BBC3 could reverse the decreased apoptotic rates induced by HOTAIR knockdown. Collectively, HOTAIR promoted mechanical stimulation-induced apoptosis by regulating the miR-221/BBC3 axis in C28/I2 cells.

## Graphic abstract

**Figure:**
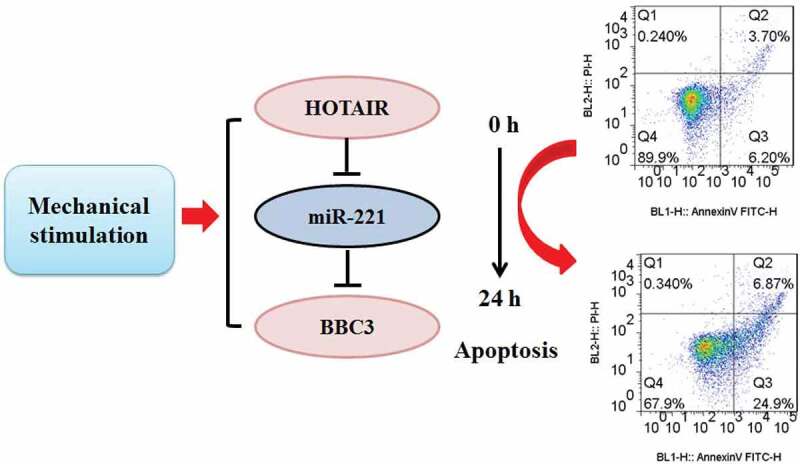

## Introduction

Mechanical loading exhibits a critical role in mediating the development and maintenance of articular cartilage. Mechanical loading is dynamic, and it is supposed to support the synthesis of extracellular matrix (ECM) under normal mechanical loading (0.4–2 MPa) [1] and inhibit the production of ECM under persistent improper loading [2]. The biomechanical stress could induce the deformation of articular cartilage, which is a thin layer tissue with low friction and acts as a cushion for sensing the body weight and exercise. Disruption of cartilage homeostasis may cause its degeneration, leading to the development of osteoarthritis (OA) [3]. Articular cartilage is a deficiency of regenerative capacity when subjected to acute or long-term abnormal mechanical stimulation. Under such circumstances, articular cartilage is susceptible to develop degenerative lesions and OA pathology [2].

Various factors contribute to mechanical stimulation-mediated OA development [1], but the precise roles still need to be further elucidated. NF-κB- and MAPK-mediated inflammatory responses are significantly enhanced by overloading [4]. Wnt/β-catenin signaling is also activated by overloading, leading to up regulation of matrix metalloproteinases (MMPs) activity and degradation of extracellular matrix (ECM) [5]. Transforming growth factor-β (TGF-β) maintains functions of articular cartilage by transducing Smad2/3 signals, thereby promoting collagen (Col2a1) and fibronectin synthesis and inhibiting ECM degradation induced by overloading [6]. However, the pathogenic development of OA induced by mechanical stimulation is still under investigation [7]. Long noncoding RNA (lncRNA) is a group of RNA molecules with more than 200 nucleotides, which cannot be translated into proteins due to a lack of an open reading frame [7,8]. Many differentially expressed lncRNAs have been involved in the pathological changes of OA [9]. LncRNA H19 has been demonstrated to ameliorate the mechanical force-induced cartilage degeneration in developmental dysplasia of the hip by mediating the miR-485/Dusp5 axis [10]. The TMSB4 pseudogene, lncRNA MSR, has been reported to be up regulated in chondrocytes in response to mechanical stress [11]. LncRNA HOX transcript antisense RNA (HOTAIR) has been demonstrated to increase osteoarthritis progression via modulation of Wnt/β-catenin signaling [12]. HOTAIR promotes lipopolysaccharide (LPS)-treated chondrocytes inflammation and apoptosis [13]. However, the roles of HOTAIR in mechanical stimulation-treated chondrocytes are still unclear. In this study, we mainly investigated the mechanism of HOTAIR on the mechanical stimulation-induced chondrocyte apoptosis.

## Materials and methods

### General information

The experimental protocol has been approved by the Ethics Committee of The First Affiliated Hospital of Gannan Medical University.

### Cell culture

Human chondrocytic C28/I2 cell line was obtained from Procell (Wuhan, China). RPMI-1640 medium (Gibco, Waltham, MA, USA) containing 10% FBS (Invitrogen, Carlsbad, CA, USA) and 100 U/mL of penicillin (Invitrogen) and streptomycin (Invitrogen) was employed for cell culture. All cells were cultured in a humidified incubator containing 5% CO_2_ at 37°C.

### Cyclic mechanical stimulation application

C28/I2 cells at 80% confluence cultured on the BioFlex plates (Flexcell Int. Co., Hillsborough, NC, USA) were transferred to a Flexcell Tension System (FX-4000) (Flexcell) with 20% surface elongation at a frequency of 6 cycles/min. Cells were harvested after 6 h, 12 h, and 24 h, respectively [14].

### MTT assays

The mechanical stimulated C28/I2 cells (2 × 10^4^ cells/well) (collection after 6 h, 12 h, and 24 h of stimulation) were cultured in 96-well plates at 37°C for 24 h. The viability of C28/I2 cells was evaluated by detecting the conversion of MTT to the formazan product, according to the instructions of kits (Cat.no.C0009S; Beyotime, Shanghai, China). The wavelength 490 nm was used for measurement by using the microplate reader (Thermo Fisher Scientific).

### Cell transfection

The short hairpin RNAs (shRNAs) HOTAIR (5’-AAAUCCAGAACCUCUGACAUUUGC-3’) and a scrambled shRNA control (sh-NC) (5’-CAUAGUCGAAUUCGCUAGUGAGUU-3’) were obtained from Shanghai GeneChem Co., Ltd. They (100 nM) were inserted into pGPH1/Neo (40 nM; GenePharma, Shanghai, China). Then, 75 pmol of constructed pGPH1/Neo were transfected into C28/I2 cells by using lipofectamine 3000 (Invitrogen), according to the instructions of the kit. Simply, the plasmid pGPH1/Neo/shRNA and lipofectamine 3000 were diluted with Opti-MEM (serum-reduced medium) and incubated for 5 min at room temperature. The two transfection mixtures were mixed and incubated at room temperature for 20 min. the transfection mixture was then gently added to each well and incubated at 37°C. Cells were transfected with sh-HOTAIR or sh-NC for 48 h prior to further investigation. Neomycin (400 μg/μL) (Sigma-Aldrich, Cat.no.1405–10-3) was added after transfection and used to select the stable transfectants [15].

MiR-221 mimics (5’-UUCCCUUGUCAUCCUUCGCCU-3’) and microRNA negative controls (miR-NC) (5’-CAGUACUUUGUAGUACAAA-3’) were purchased from RiboBio (Guangzhou, China). C28/I2 cells at the confluence of 60% (1 × 10^5^ cells/well or about 1 × 10^4^ cells/cm^2^) were conducted for transfection by using lipofectamine 3000 (Invitrogen) according to the kits instructions, after they were cultured for one day in the 6-well plates. The designed concentrations of miR-221 mimics and miR-NC in the final transfection system were all 50 nM [16].

Furthermore, cloning the open reading frame of Bcl-2 binding component 3 (BBC3) into the pcDNA3.1 vector is prepare to develop the pcDNA3.1-BBC3 vector (RiboBio). Next, pcDNA3.1-BBC3 vectors were transfected into C28/I2 cells by using lipofectamine 3000, according to the instructions of the kit (RiboBio). The transfected cells were incubated with 5% CO_2_ at 37°C, and 48 h after transfection for the further investigation

### Apoptosis determination

Flow cytometry (FACSCalibur BD, San Jose, CA, USA) was used for determining the apoptotic changes by using Annexin V-FITC apoptosis assay, according to the instructions recommended by the kits. Simply, C28/I2 cells were harvested and incubated in the buffer containing Annexin V-FITC and PI, respectively. The apoptotic changes of C28/I2 cells were determined.

### qRT-PCR

Trizol reagent (Invitrogen) was used for extraction of total RNA from C28/I2 cells under the guideline of the kit instructions. Specifically, RNA (2 μg) was reverse transcribed to cDNA using M-MLV (Promega, Madison, WI, USA). Quantitative PCR assays were conducted on Power SYBRs Green PCR Master Mix (Applied Biosystems, CA, USA) to detect the expression of HOTAIR, BBC3, and caspase-3. The expression of miR-221 was detected using the Taqman MicroRNA Reverse Transcription Kit and Taqman Universal Master Mix II kit (Applied Biosystems). GAPDH and U6 were used as the endogenous reference for mRNA and miRNA, respectively. All the primers for the sense and anti-sense chains were obtained from Biomics. The primer sequences are listed as follows: HOTAIR forward: 5’-TAGGCAAATGTCAGAGGGTT-3’, reverse: 5’-ACACAAGTAGCAGGGAAAGG-3’; BBC3 forward: 5’-TTGTGCTGGTGCCCGTTCCA-3’, reverse: 5’-AGGCTAGTGGTCACGTTTGGCT-3’; caspase-3 forward: 5’-TTTGTTTGTGTGCTTCTGAGCC-3’, reverse: 5’-ATTCTGTTGCCACCTTTCGG-3’; GAPDH forward: 5’-AGGTGAAGGTCGGAGTCAACG-3’, reverse: 5’-AGGGGTCATTGATGGCAACA-3’; miR-221 forward: 5’-GGGAAGCTACATTGTCTGC-3’, reverse: 5’-CAGTGCGTGTCGTGGAGT-3’; U6 forward: 5’-CTCGCTTCGGCAGCACA-3’, reverse: 5’-AACGCTTCACGAATTTGCGT-3’. The gene expression of miRNA and mRNA was indicated as fold changes by employing 2^−ΔΔCT^ method [17].

### Western blotting

The total proteins were extracted from cultured cells in ice-cold RIPA lysis buffer (Beyotime), and the protein concentrations were determined by using a BCA protein assay kit (Beyotime). 30 μg total proteins of each experimental group were subjected to 10% sodium dodecyl sulfate-polyacrylamide gel electrophoresis (SDS-PAGE) and then transferred onto polyvinylidene fluoride (PVDF) membranes (Millipore, Burlington, MA, USA). After being blocked in tris-buffered saline (TBS) containing 5% nonfat milk for 1 h at room temperature, the membranes were incubated with the primary antibodies at 4°C overnight against BBC3 (1:1,000 dilutions, Cat.no.SAB3500464, Sigma), caspase-3 (1:1,000 dilutions, Cat.no.C8487, Sigma), and GAPDH (1:1,000 dilutions, Cat.no.SAB1410512, Sigma). Then, the membranes were incubated with the secondary antibody conjugated with peroxidase (1:2,000 dilutions, Cat.no. AP510, Sigma) for 1 h. Protein bands were detected by using the enhanced chemiluminescence detection system (Bio-Rad, Hercules, CA, USA) and Quantity One software v4.6.2 (Bio-Rad). The quantification of protein expression was compared to that of GAPDH.

### Dual-luciferase reporter assay

The online predicted system StarBase v2.0 (http://starbase.sysu.edu.cn) and TargetScan7.2 (http://www.targetscan.org) were employed to seek the miRNA target of HOTAIR and target gene for miR-221, respectively. The recombinant luciferase plasmids were constructed by cloning the sequences of wild-type (WT) HOTAIR and 3’-UTR of BBC3, respectively, into the pGL-3 luciferase basic vector (Promega). In addition, their mutant-types with mutant binding sites for miR-221 were also constructed as MUT-HOTAIR and MUT-BBC3, respectively. Each constructed plasmid was transfected into C28/I2 cells with miR-221 mimics or miR-NC by using lipofectamine 3000 (Invitrogen). Following incubation for 48 h at 37°C, firefly and Renilla luciferase activities were detected by using the Glomax 96 luminometer (Promega) according to the instructions of the kits. Firefly luciferase reporter was normalized to Renilla luciferase activity [18].

### RNA immunoprecipitation (RIP) assays

RIP assays were conducted to further investigate the direct interaction between HOTAIR and miR-221 by employing a Magna RNA immunoprecipitation kit (EMD Millipore), according to the manufacturer’s instructions. C28/I2 cells (2 × 10^7^ cells) were lysed and then incubated with magnetic beads, which are pre-coated with antibodies against Argonaute2 (Ago2; Cat.no.MABE56, Sigma-Aldrich) using anti-immunoglobulin G (IgG; Cat.no.I5131, Sigma-Aldrich) as the negative control. The RNA was extracted and detected by qRT-PCR. Finally, the levels of HOTAIR and miR-221 in anti-IgG and anti-Ago2 groups were compared [19].

### Statistical analysis

All experiments were performed in triplicate and data are presented as the mean ± standard error of the mean. SPSS 20.0 software (IBM Corp., Armonk, New York, USA) was used for statistical analysis. One-way ANOVA and Tukey’s post hoc test were used to compare differences between multiple groups. P < 0.05 was considered to indicate a statistically significant difference.

## Results

### Mechanical stimulation promoted cell apoptosis and up regulated HOTAIR/miR-221 expression

**Figure 1.**
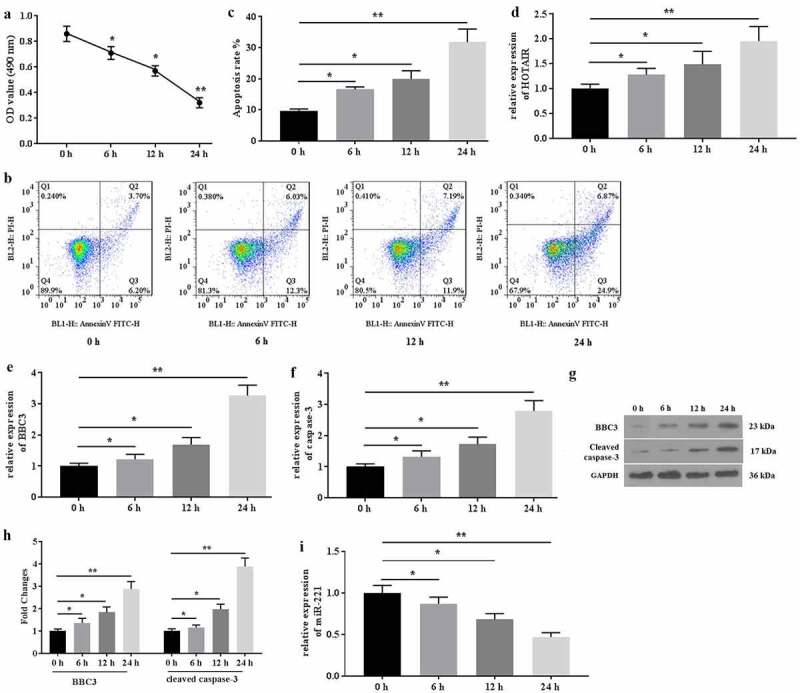
Mechanical stimulation induced apoptosis and mediated the expression of HOTAIR/miR-221 in C28/I2 cells. (a) The MTT assays were conducted after 0, 6 h, 12 h, and 12 h of mechanical stimulation. (b) The apoptosis was determined by flow cytometer, and the apoptotic rates (c) were calculated. The genes expression of HOTAIR (d), BBC3 (e), and caspase-3 (f) were detected by qRT-PCR. (g) The proteins expression of BBC3 and cleaved caspase-3 were determined by western blot. (h) The fold changes of protein expression were calculated. (i) The expression of miR-221 were detected by qRT-PCR. All experiments were performed in triplicate and data are presented as the mean ± standard deviation. *P < 0.05 and **P < 0.01

The expression of HOTAIR and the cell apoptosis in mechanical stimulation-treated chondrocytes were detected. The results from MTT assays showed that mechanical stimulation (20% surface elongation at a frequency of 6 cycles/min) significantly decreased the viability of C28/I2 cells in a time-dependent manner (Figure 1(a)). Similarly, a study from the flow cytometer indicated that mechanical stimulation deteriorated cell apoptosis (Figure 1(b,c)). In addition, mechanical stimulation up regulated the expression of HOTAIR (Figure 1(d)), BBC3 (Figure 1(e-h)), and caspase-3 (Figure 1(f-h)) and down regulated the expression of miR-221 (Figure 1(i)) in C28/I2 cells. Collectively, mechanical stimulation promoted cell apoptosis, which might be associated with aberrant expression of HOTAIR/miR-221 in C28/I2 cells.

### Knockdown of HOTAIR ameliorated mechanical stimulation-induced cell apoptosis

**Figure 2.**
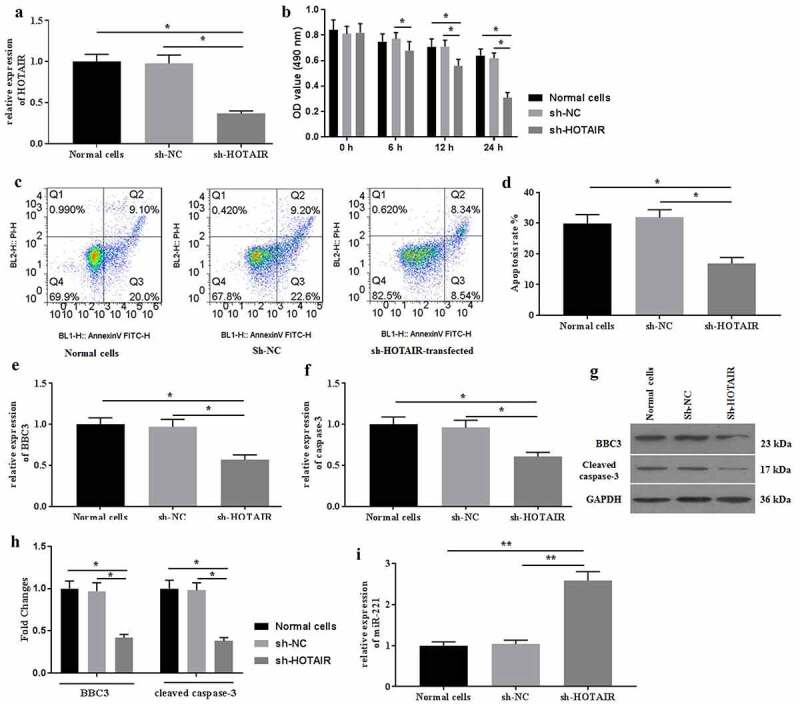
Knockdown expression of HOTAIR ameliorated cell apoptosis induced by mechanical stimulation. (a) The expression of HOTAIR was determined by qRT-PCR. (b) The cell viability was detected by MTT assays after transfection of sh-HOTAIR. (c) The apoptosis was determined by flow cytometer, and the apoptotic rates (d) were calculated. The mRNA expression of BBC3 (e), caspase-3 (f), and miR-221 (i) were determined by qRT-PCR after transfection of sh-HOTAIR. The protein expression of BBC3 (g and h) and cleaved caspase-3 (**G** and **H**) were determined by western blot. All experiments were performed in triplicate and data are presented as the mean ± standard deviation. *P < 0.05 and **P < 0.01

Then, the effects of HOTAIR knockdown on mechanical stimulation-induced cell apoptosis were determined. To investigate the roles of HOTAIR in mechanical stimulation-induced cell apoptosis, sh-HOTAIR was established and transfected into C28/I2 cells. The expression of HOTAIR was detected by qRT-PCR for identification of successful transfection (Figure 2(a)). Transfection of sh-HOTAIR improved cell viability (Figure 2(b)). To provide a more distinguishable difference, the following experiments were conducted under mechanical stimulation for 24 h. As a result, sh-HOTAIR-transfection significantly ameliorated cell apoptosis (Figure 2(c,d)) induced by mechanical stimulation. Moreover, the expression of BBC3 (Figure 2(e-h)), caspase-3 (Figure 2(f-h)), and miR-221 (Figure 2(i)) were also reversed, compared with those in the non-transfected group. Collectively, mechanical stimulation-induced cell apoptosis by regulating the expression of HOTAIR signaling in C28/I2 cells.

### HOTAIR interacted with miR-221

**Figure 3.**
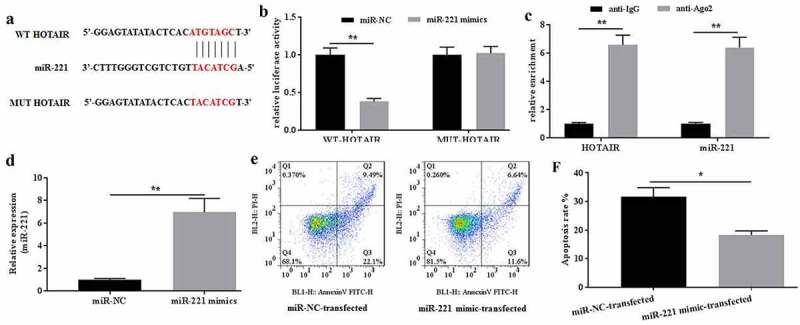
MiR-221 interacted with HOTAIR and inhibited its effects on apoptosis induced by mechanical stimulation. (a) The potential interaction between HOTAIR and miR-221 was predicted by Starbase2.0 software. (b) The relative luciferase activity was detected in C28/I2 cells co-transfected with both WT/MUT-HOTAIR and miR-221 mimics/miR-NC. Firefly luciferase reporter activity was normalized to Renilla luciferase activity. (c) The interaction between HOTAIR and miR-221 was confirmed by RIP assays. (d) The expression level of miR-221 in miR-221 mimic-transfected C28/I2 cells was determined by qRT-PCR. (e) The apoptosis was determined by flow cytometer, and the apoptotic rates (f) were calculated. All experiments were performed in triplicate and data are presented as the mean ± standard deviation. *P < 0.05 and **P < 0.01

The interaction between HOTAIR and miR-221 was verified. To further explore the roles of HOTAIR in mechanical stimulation-treated C28/I2 cells, the potential miRNAs that bind to HOTAIR were explored by the predicting software Starbase2.0. As a result, miR-221 might be a potential target of HOTAIR (Figure 3(a)), and the location of miR-221 on HOTAIR was chr12:54356181–54356203. The dual-luciferase reporter assays showed that the luciferase activity in the reporter containing the WT-HOTAIR decreased by more than 60%. In contrast, no significant differences were observed in the relative luciferase activities between the NC reporter and the reporter containing the MUT-HOTAIR (Figure 3(b)). In addition, RIP assays also indicated that HOTAIR could interact with miR-221 (Figure 3(c)). Taken together, miR-221 could be the potential target of HOTAIR.

### Overexpression of miR-221 abrogated the effects of HOTAIR on mechanical stimulation-treated C28/I2 cells

The effects of miR-221 on HOTAIR-mediated apoptosis in mechanical stimulation-treated chondrocytes were determined. To investigate the possible roles of miR-221 in mechanical stimulation-treated C28/I2 cells, miR-221 mimics were transfected into C28/I2 cells. The expression of miR-221 was determined for the identification of successful transfection (Figure 3(d)). After mechanical stimulation for 24 h, flow cytometer study was explored and showed that miR-221 mimics could effectively ameliorate the apoptotic rate induced by mechanical stimulation (Figure 3(e,f)). Thus, overexpression of miR-221 could reverse the effects of HOTAIR on mechanical stimulation-induced apoptosis in C28/I2 cells.

### BBC3 was a direct target of miR-221

**Figure 4.**
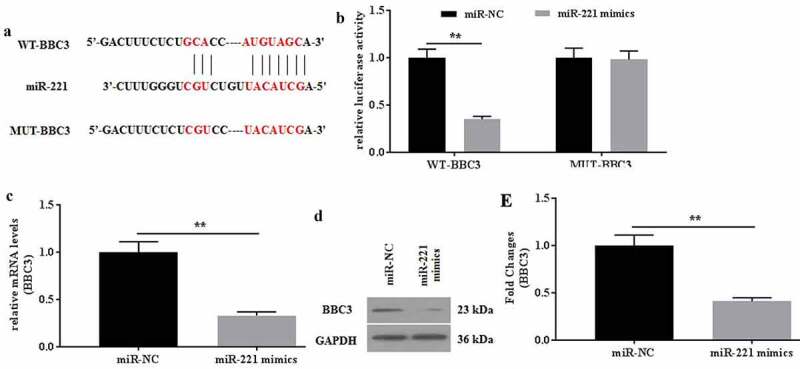
BBC was identified as a direct target of miR-221. (a) The potential interaction between miR-221 and BBC3 was predicted by TargetScan7.2 software. MiR-221 bound to the position 96–103 of BBC3 3’-UTR. (b) The relative luciferase activity was detected in C28/I2 cells co-transfected with both WT/MUT-BBC3 and miR-221 mimics/miR-NC. The mRNA (c) and protein (d-e) expression of BBC was detected in miR-221/miR-NC-transfected C28/I2 cells. All experiments were performed in triplicate and data are presented as the mean ± standard deviation. **P < 0.01

The interaction between miR-221 and BBC3 was verified. To further explore the roles of miR-221 in C28/I2 cells, the target genes of miR-221 were predicted by TargetScan7.2. As a result, BBC3 might be the potential target of miR-221 (Figure 4(a)), which is verified by the dual-luciferase reporter assay (Figure 4(b)). The relative luciferase activities did not show a statistical difference between the NC reporter and the reporter containing the mutant site of BBC3. In contrast, the relative luciferase activities in the reporter containing the WT binding site of BBC3 decrease by more than 60%. The mRNA and protein expression of BBC3 was determined. It found that miR-221 mimics could significantly down regulate the expression of BBC3 mRNA (Figure 4(c)) and protein (Figure 4(d-e)). Collectively, miR-221 might specifically target to degrade BBC3 by binding to its 3’-UTR.

### Overexpression of BBC3 rescued the effects induced by HOTAIR knockdown on mechanical stimulation-treated C28/I2 cells

**Figure 5.**
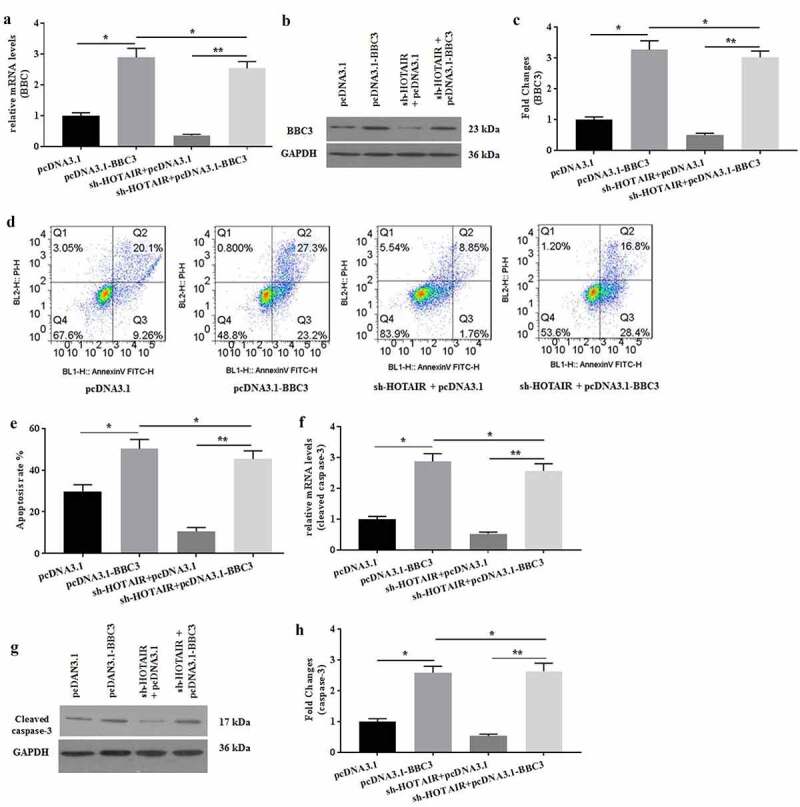
Overexpression of BBC3 reversed the effects of HOTAIR knockdown expression. The mRNA (a) and protein (b-c) expression of BBC3 was detected by pRT-PCR and western blot, respectively. (d) The cell apoptosis was detected by flow cytometer, and the apoptotic rates were calculated (e). The mRNA (f) expression of caspase-3 and the protein (g-h) expression of cleaved caspase-3 were detected by pRT-PCR and western blot, respectively. All experiments were performed in triplicate and data are presented as the mean ± standard deviation. *P < 0.05 and **P < 0.01

The effects of BBC3 on HOTAIR-mediated apoptosis in mechanical stimulation-treated chondrocytes were determined. To further explore the roles of BBC3 in HOTAIR-mediated C28/I2 cells under mechanical stimulation, pcDNA3.1-BBC3 was prepared for co-transfection with sh-HOTAIR into C28/I2 cells. The mRNA (Figure 5(a)) and protein (Figure 5(b-c)) expression of BBC3 were detected to verify the successful co-transfection. Overexpression of BBC3 reversed the decreased apoptosis of C28/I2 cells induced by HOTAIR knockdown (Figure 5(d-e)). Similarly, overexpressed BBC3 also enhanced the expression of caspase-3 (Figure 5(f-h)), which was attenuated by sh-HOTAIR transfection in C28/I2 cells. Collectively, overexpression of BBC3 might rescue the effects of HOTAIR knockdown on C28/I2 cells.

## Discussion

Abnormal mechanical stimulation associated with obesity, trauma, and joint instability has been demonstrated to change joint loading and be closely related to chondrocytes apoptosis and cartilage degeneration [20,21]. However, the underlying mechanism of mechanical stimulation in mediating chondrocytes apoptosis and cartilage degeneration is still unclear. It is necessary to explore the roles of mechanical stimulation in OA pathological development. In this article, we mainly found that mechanical stimulation (20% surface elongation at a frequency of 6 cycles/min) promoted apoptosis in C28/I2 cells by up regulating the expression of HOTAIR, which sponged miR-221. Knockdown expression of HOTAIR ameliorated cell apoptosis induced by mechanical stimulation. BBC3 was identified as a direct target of miR-221. Overexpression of miR-221 could effectively rescue the decreased apoptosis induced by HOTAIR knockdown in C28/I2 cells.

Many studies have demonstrated that bulk RNA sequencing and single-cell sequencing data have been used for the analysis in the pathological development of OA [21,22]. Sphingosine kinase 1 (SPHK1), a member of the SPHK family, is associated with angiogenesis and promotes the survival of endothelial cells, the processes of cartilage degradation, and the development of OA [23]. LncRNA LINC00917 and CTD-2246P4.1 have been reported to regulate angiogenesis in OA cartilage by mediating SPHK1 [24]. LncRNA GAS5 expression is up regulated in OA cartilage tissues. Silence of GAS5 increases the autophagy ability and decreases the apoptosis rate by sponging miR-144 [25]. In addition, the increased expression of lncRNA PVT1 in diabetic OA cartilage is also associated with Mankin score and reduced expression of type II collagen by negatively interacting with miR-146a, increasing the productions of inflammatory cytokines, and activating TGFβ/SMAD4 signaling pathway [26]. In our study, we found that HOTAIR was up regulated by mechanical stimulation in C28/I2 cells. Knockdown of HOTAIR expression could ameliorate cell apoptosis induced by mechanical stimulation.

MicroRNAs suppress genes expression post-transcriptionally. Dysregulation of microRNAs in osteoarthritis has been reported [27]. MicroRNAs have been shown to play roles in chondrocyte phenotype through signaling pathways, apoptosis, autophagy, and senescence [28]. MiR-29 acts across the development and progression of OA by negatively regulating Smad, NF-κB, and canonical Wnt signaling pathways [29]. MiR-34a, miR-146a, and miR-181a have been the possible mediator of hydrostatic pressure effects on oxidative stress in OA chondrocytes. Silencing of miR-34a, miR-146a, and miR-181a significantly down regulates the expression of MMP-13 and ADAMTS-5 and up regulates the expression of Col2a1, mediating the effects of hydrostatic pressure on chondrocytes apoptosis [30]. BBC3 is a member of the Bcl-2 family and belongs to the BH3-only pro-apoptotic subclass. BBC3 can interact with Bcl-2 family members to induce mitochondrial outer membrane permeabilization and apoptosis [31]. In our study, miR-221 expression was down regulated, and overexpression of miR-221 could effectively attenuate cell apoptosis induced by mechanical stimulation in C28/I2 cells. The expression of BBC3 is up regulated by mechanical stimulation. Overexpression of BBC3 dramatically increased apoptotic rates induced by mechanical stimulation, reversing the effects of HOTAIR knockdown in C28/I2 cells. In addition, BBC3 was identified as the direct target of miR-221.

Recently, it has been demonstrated that HOTAIR can promote chondrocytes apoptosis by activating Wnt/β-catenin signaling [12,32]. In addition, HOTAIR can specifically bind to miR-221 [33]. Consistently, our study also verified this interaction. Another study showed that miR-221 can inactivate Wnt/β-catenin signaling by targeting to degrade DKK2, which is a receptor for activation of Wnt/β-catenin signaling [34]. It has been shown that Wnt/β-catenin signaling exhibits a regulatory activity on the expression of BBC3 in lung cancer cells [35]. It is reported that β-catenin can down regulate the expression of BBC3 by increasing the expression of miR-483, which targets to degrade BBC3 in HepG2 cells [36]. The biological effects of Wnt/β-catenin signaling on cell apoptosis are associated with the expression of BBC3 multiple myeloma cells [37]. Whether the apoptotic effects of HOTAIR/miR-221/BBC3 is associated with the activity of Wnt/β-catenin signaling in mechanical stimulation-induced chondrocytes apoptosis is still needed to be elucidated.

## Conclusion

Collectively, mechanical stimulation (20% surface elongation at a frequency of 6 cycles/min) could promote apoptosis by up regulating the expression of HOTAIR through sponging miR-221 in C28/I2 cells.

## Data Availability

All data generated or analyzed during this study are included in this published article.

## Highlights

(1) The expression of HOTAIR in C28/I2 cells was significantly up regulated by mechanical stimulation

(2) Up regulation of HOTAIR promoted apoptotic rate in mechanical stimulation-treated C28/I2 cells.

(3) HOTAIR enhanced mechanical stimulation-induced apoptosis by increasing the expression of BBC3 through sponging miR-221 in C28/I2 cells.
